# SYK Inhibition Induces Apoptosis in Germinal Center-Like B Cells by Modulating the Antiapoptotic Protein Myeloid Cell Leukemia-1, Affecting B-Cell Activation and Antibody Production

**DOI:** 10.3389/fimmu.2018.00787

**Published:** 2018-04-24

**Authors:** Nathalie Roders, Florence Herr, Gorbatchev Ambroise, Olivier Thaunat, Alain Portier, Aimé Vazquez, Antoine Durrbach

**Affiliations:** ^1^Institut Francilien de Recherche en Nephrologie et Transplantation (IFRNT), Service de Néphrologie, Hôpital Bicêtre, Le Kremlin Bicêtre, France; ^2^INSERM UMRS-MD 1197, Villejuif, France; ^3^Université Paris Sud, Orsay, France; ^4^French National Institute of Health and Medical Research (INSERM) Unit 1111, Lyon, France; ^5^Department of Transplantation, Nephrology and Clinical Immunology, Edouard Herriot University Hospital, Lyon, France; ^6^Claude Bernard University Lyon 1, Lyon, France

**Keywords:** germinal center B-cells, spleen tyrosine kinase inhibition, myeloid cell leukemia-1, apoptosis, antibody-mediated rejection

## Abstract

B cells play a major role in the antibody-mediated rejection (AMR) of solid organ transplants, a major public health concern. The germinal center (GC) is involved in the generation of donor-specific antibody-producing plasma cells and memory B cells, which are often poorly controlled by current treatments. Myeloid cell leukemia-1 (Mcl-1), an antiapoptotic member of the B-cell lymphoma-2 family, is essential for maintenance of the GC reaction and B-cell differentiation. During chronic AMR (cAMR), tertiary lymphoid structures resembling GCs appear in the rejected organ, suggesting local lymphoid neogenesis. We report the infiltration of the kidneys with B cells expressing Mcl-1 in patients with cAMR. We modulated GC viability by impairing B-cell receptor signaling, by spleen tyrosine kinase (SYK) inhibition. SYK inhibition lowers viability and Mcl-1 protein levels in Burkitt’s lymphoma cell lines. This downregulation of Mcl-1 is coordinated at the transcriptional level, possibly by signal transducer and activator of transcription 3 (STAT3), as shown by (1) the impaired translocation of STAT3 to the nucleus following SYK inhibition, and (2) the lower levels of Mcl-1 transcription upon STAT3 inhibition. Mcl-1 overproduction prevented cells from entering apoptosis following SYK inhibition. *In vitro* studies with primary tonsillar B cells confirmed that SYK inhibition impaired cell survival and decreased Mcl-1 protein levels. It also impaired B-cell activation and immunoglobulin G secretion by tonsillar B cells. These findings suggest that the SYK–Mcl-1 pathway could be targeted, to improve graft survival by manipulating the humoral immune response.

## Introduction

B cells play a major role in acute and chronic antibody-mediated rejection (AMR) and allograft survival. AMR after solid organ transplantation is associated with a high frequency of organ deterioration and graft loss, despite the availability of treatment. Current therapies include plasmapheresis (1, 2), high-dose intravenous immunoglobulin (3, 4), monoclonal anti-CD20 antibodies (5, 6), proteasome inhibitors (4, 7), and complement inhibitors (8, 9). However, these treatments frequently fail to control AMR, donor-specific antibody (DSA) production, and memory B-cell formation.

B-cell activation through B-cell receptor (BCR) signaling drives B-cell survival, differentiation, anergy, or apoptosis, depending on the other signals received by the cell. Antigen (Ag)-dependent BCR activation leads to the recruitment and activation of spleen tyrosine kinase (SYK) (10, 11). Active SYK induces the formation of a signalosome, containing kinases and adaptor proteins, which sets in motion signaling cascades, such as those involving AKT, mitogen-activated protein kinases, nuclear factor of activated T cells, and nuclear factor-κB (NFκB), resulting in translational modifications (12). Germinal centers (GC) play an important role in driving the differentiation of B cells into DSA-producing plasma and memory B cells (13). In solid organ transplantation, the development of tertiary lymphoid structures (TLS) sustaining a functional ectopic germinal center reaction has been demonstrated in human grafts displaying chronic AMR (cAMR) (14, 15). Following T cell-dependent B-cell activation and GC formation, B cells undergo clonal expansion, isotype class switching, somatic hypermutation (16–18), and affinity maturation and selection (19–21). They leave the GC as highly specific long-lived memory B-cells and antibody (Ab)-producing plasma cells.

The persistence of the GC and the selection of high-affinity effector B cells are regulated by pro- and antiapoptotic signals influenced by Ag binding and cell-mediated interactions (20, 22, 23). The members of the B-cell lymphoma-2 (Bcl-2) protein family maintain the delicate balance between cell survival and apoptotic death. The proteins of this family form three groups: (i) antiapoptotic proteins, including myeloid cell leukemia-1 (Mcl-1), Bcl-2, and Bcl-xs/l, which play an essential role in cell survival; (ii) proapoptotic proteins, BAX and BAK, required to trigger downstream apoptotic processes, such as cytochrome *c* release from the mitochondria, and subsequent caspase activation; (iii) the so-called “BH-3-only” proteins, including Puma, Noxa, Bad, Bid, and Bim, which interact with the other members of the family to control their activity (24–26). Vikstrom et al. (27) showed that no GCs or memory B-cells developed in the absence of Mcl-1, highlighting the importance of Mcl-1 for GC maintenance and B-cell differentiation. In a previous study of the role of PUMA in regulating mitogen-activated B cells and memory B cells, we observed that both PUMA and Mcl-1 were expressed in GCs *in vivo* (28), suggesting a possible key role in the maintenance and development of GC and memory B cells.

In kidneys displaying cAMR, we observed an infiltration of B cells expressing Mcl-1, like pre-GC and GC B cells. We investigated the relationship between BCR signaling and B-cell survival and differentiation, by inhibiting SYK. Using Burkitt’s lymphoma-derived cells as a model for GC centroblasts, we showed that SYK inhibition decreased cell viability. SYK inhibition reduced Mcl-1 gene expression *via* signal transducer and activator of transcription 3 (STAT3). Immunoglobulin (Ig) synthesis was also impaired by SYK inhibition in primary B cells *in vitro*; these cells were also less viable, less strongly activated, and had lower Mcl-1 protein levels.

## Materials and Methods

### Reagents and Antibodies

The following reagents were used: BAY61-3606 (Merck Millipore), Stattic (Torcis), Q-VD-Oph (Sigma), MG-132 (Calbiochem), and cycloheximide (Sigma).

We used the following primary Abs: hCD19-APD-Cy-7 (SJ25C1; BD Biosciences Cat# 557791 RRID:AB_396873), hCD38-BV421 (HIT2; BD Biosciences Cat# 562445 RRID:AB_11153870) hsIgD-Pe-Cy7 (IA6-2; BD Biosciences Cat# 561314 RRID:AB_10642457), hCD80-BV605 (L307.4; BD Biosciences Cat# 563315), hPhospho-SYK-AF488 (C87C1; Cell Signaling Technology Cat# 4349), and hMcl-1-FITC (Biorbyt Cat# orb15956 RRID:AB_10747574) for flow cytometry. Anti-Mcl-1 (S-19; Santa Cruz Biotechnology Cat# sc-819 RRID:AB_2144105), anti-Bcl-2 (C-2; Santa Cruz Biotechnology Cat# sc-819 RRID:AB_2144105), anti-Bcl-x_S/L_ (S-18; Santa Cruz Biotechnology Cat# sc-819 RRID:AB_2144105), anti-GAPDH (Sigma-Aldrich Cat# G9545 RRID:AB_796208), and anti-PARP (Cell Signaling Technology Cat# 9542 also 9542S, 9542L, 9542P RRID:AB_2160739) antibodies were used for immunoblotting and anti-hCD19 (HIB19; BD Biosciences Cat# 555409 RRID:AB_395809), anti-hMcl-1 (Sigma-Aldrich Cat# HPA008455 RRID:AB_1079334), and anti-STAT3 (K-15; Santa Cruz Biotechnology Cat# sc-483 RRID:AB_632441) Abs were used for immunohistology.

Retroviral particles were generated with the following plasmids: the expression plasmid pBabe-Flag-hMcl-1, a gift from Roger Davis (29) (Addgene plasmid # 25371), the empty vector control plasmid pBabe-puro-IRES-EGFP, a gift from L. Miguel Martins (Addgene plasmid # 14430), the envelope-expressing plasmid pCMV-VSV-G, a gift from Bob Weinberg (Addgene plasmid # 8454), and the packaging plasmid pCL-Eco (Novus Biologicals).

### Cell Culture

BL41, RAMOS, and BL2 Burkitt’s lymphoma cells were cultured in complete RPMI medium: RPMI-1640 (Sigma) supplemented with 10% heat-inactivated fetal bovine serum (FBS, Dominique Dutscher), 100 U/ml penicillin and 100 µg/ml streptomycin (Sigma).

HEK 293T and CD40 ligand/CD32 ligand-expressing murine fibroblasts were cultured in complete Dulbecco’s modified Eagle medium (DMEM; Sigma) supplemented with 10% FBS, 100 U/ml penicillin, 100 µg/ml streptomycin, and 0.1 mg/ml Normocin™ (InvivoGen).

Renal biopsy specimens and detransplanted kidneys were obtained from transplant recipients presenting end-stage renal failure due to cAMR, who gave written informed consent, in accordance with the Helsinki Declaration, for the use of part of their biopsy specimens for research on renal transplantation. The samples were stored at the Biological Resource Center of Paris-South University. This collection has been declared to the French Ministry of Research: AC-2017-2991.

Cells were isolated from detransplanted kidneys by separating the cortex from adipose tissue and the medulla. The tissue was dissected and added to enzyme buffer (70% DMEM, 1% BSA, 2.4 mM CaCl_2_, 0.4 mg/ml liberase, and 240 U DNase) and dissociated with a GentleMACS™ Dissociator (Miltenyi Biotech). The cell suspension was incubated for 45 min at 37°C, with shaking. Cells were separated from the tissue with a stainless-steel strainer and a glass grinder. Cells were washed with HEPES Eagle’s medium buffer (70% DMEM, 1% BSA, 2.4 mM CaCl_2_,). Mononuclear cells were isolated from the cell suspension by centrifugation on a Pancoll (Human; PAN™ Biotech) gradient (2:1 ratio of diluted cells to Pancoll) at 600 × *g* for 20 min. Mononuclear cells were isolated and washed in complete RPMI.

Primary cells were isolated from tonsillar tissue removed from patients during tonsillectomy. The tonsils were dissected and pushed through a stainless-steel strainer with a glass grinder. Cells were collected and washed with complete RPMI, then homogenized by passage through a nylon cell strainer with 100 µm pores (BD Bioscience).

Tonsillar cells were cocultured in complete RPMI, with CD40 ligand/CD32 ligand-expressing fibroblasts, in the presence of anti-μ Abs (Jackson Immunoresearch), LPS (Sigma), and BAY61-3606. Fibroblast growth was blocked by incubation with mitomycin C (10 µg/ml, Roche) for 30 min at 37°C, under an atmosphere containing 5% CO_2_, before the addition of tonsillar cells.

### Retrovirus Production and Cell Transduction

Retroviruses were generated by the transient cotransfection of HEK 293T cells with a three-plasmid combination, by the calcium phosphate coprecipitation method, as previously described (30). After 3 days of culture in cells, the retroviral particles were collected and concentrated with 5× PEG IT™ viral precipitation solution (System Biosciences).

For retroviral transduction, 2 × 10^6^ BL41 cells were collected and mixed with a suspension of retroviral particles in the presence of Polybrene (Santa Cruz Biotechnology). The resulting suspension was then centrifuged at 300 × *g* for 90 min. Cells were incubated at 37°C under an atmosphere containing 5% CO_2_ for 2 h, and fresh complete RPMI was then added. Transduced cells were selected by a series of puromycin (1 µg/ml; InvivoGen) treatments.

### Western Blotting

Whole-cell lysates were prepared in lysis buffer [50 mM Tris–HCl, pH 7.4, 150 mM NaCl, 2 mM EDTA (ethylenediaminetetraacetic acid), 1% Triton X-100, and 1% Igepal/NP-40, supplemented with Halt™ Protease inhibitor cocktail (Thermo Scientific)]. For phosphorylation analysis, the phosphatase inhibitors ß-glycerophosphate (12.5 nM), sodium orthovanadate (10 µM), sodium fluoride (0.1 mM), and *N*-ethylmaleimide (30 µM) were added before cell lysis. Protein determinations were performed with the micro-BCA protein assay kit (Thermo Scientific). Protein samples (equal masses) were heated for 5 min at 99°C after the addition of Tris–glycine SDS sample buffer (Life Technologies) containing 10% ß-mercaptoethanol (Sigma). They were subjected to polyacrylamide gel electrophoresis and the protein bands were transferred onto nitrocellulose membranes (Santa Cruz). The membranes were incubated with primary Abs, and Ab binding was visualized by chemiluminescence with horseradish peroxide (HRP)-conjugated secondary Abs (Jackson Immunoresearch), the Immobilon HRP chemiluminescence substrate for western blots (Millipore) and a DDC camera (LAS-4000 mini, Fujifilm).

### Flow Cytometry

We assessed the viability of BL41, RAMOS, and BL2 cells by flow cytometry, with a BD Accuri C6 flow cytometer (BD Biosciences). Viability was assessed by expressing the proportion of viable cells (excluding granular and shrunken cells) as a percentage of the total cell population, based on forward (fw) and side light scattering profiles.

Apoptotic cells were identified with the Pacific Blue Annexin V Apoptosis detection kit and 7-AAD (Biolegend), according to the manufacturer’s protocol. Cells were analyzed in a BD LSRFortessa flow cytometer (BD Biosciences).

For extracellular staining, cells were incubated with fvs620 (1:1,000 dilution; BD Biosciences) in 1× PBS for 15 min at room temperature. Non-specific binding was blocked with human BD FC block (2.5 μg/1 × 10^6^ cells; BD Biosciences) in 1× PBS supplemented with 2.5% FCS and 0.1% sodium azide, and cells were then incubated with fluorescent Abs. Intracellular staining for non-phosphorylated proteins was performed as follows: cells were fixed in 4% paraformaldehyde (PFA, Alfa Aesar), then incubated with NH_4_Cl (100 mM) for quenching, permeabilized with 0.15% saponin (VWR), and incubated with conjugated Abs in the presence of saponin.

For intracellular staining for phosphorylation analysis, saponin was replaced with 0.1% Triton X-100 (Sigma) and 50% methanol was added, and the cells were then left on ice for permeabilization. The cells were then incubated with fluorochrome-conjugated Abs and analyzed in a BD LSRFortessa flow cytometer (BD Biosciences). All flow cytometry data were analyzed with FlowJo^tm^ (FlowJo Treestar, RRID:SCR_008520) software.

### Enzyme-Linked Immunosorbent Assay

Total immunoglobulin G (IgG) secretion by tonsillar B cells was assessed with the Human IgG Total ELISA Ready-SET-Go!^®^ kit (Affymetrix, eBioscience) according to the manufacturer’s protocol, with undiluted supernatant. Absorbance was read at 450 and 570 nm, with a FLUOstar Omega microplate reader (BMG Labtech). Secreted IgG was quantified by comparison with a standard curve.

### Immunofluorescence

The paraffin was removed from the sections by three sequential washes in xylene (Sigma). The sections were then rehydrated by passage through a series of ethanol solutions in water (100, 90, and 70% ethanol in distilled water). Ags were retrieved by boiling samples twice, for 5 min each, in citrate buffer (pH 6) supplemented with 0.05% Tween-20 (Sigma) in a microwave oven, and allowing the sample to cool to room temperature. Non-specific binding to FC receptors was blocked by incubating slides for 1 h at room temperature in blocking buffer (1× PBS, 1% FBS, 1% BSA, 1% human AB serum) supplemented with 0.1% Triton X-100, and then incubating them with primary Abs followed by AF488- or AF594-conjugated secondary Abs (Life Technologies).

BL41 cells were attached to poly-l-lysine slides (Thermo Scientific), fixed in 4% PFA and permeabilized by incubation with 0.15% Triton X-100. Samples were blocked by incubation with 10% FBS in 1× PBS and quenched with NH_4_Cl (100 mM). Samples were incubated with primary Abs and then with AF488-conjugated secondary Abs (Life Technologies).

Nuclei were stained with DAPI (4,6 diamidino-2-phenylindole, 1:10,000; Life Technologies) and the sections were mounted on slides in Fluoromount-G™ slide-mounting medium (Beckman Coulter) and covered with a coverslip.

Images were acquired with a Leica SP5 confocal microscope (Leica Microsystems) equipped with a 63× oil immersion fluorescence objective.

### Gene Expression Analysis

RNA was extracted from BL41, RAMOS, and BL2 cells with the RNeasy plus mini kit (Qiagen), according to the manufacturer’s protocol, including lysate homogenization with QIAshredder spin columns (Qiagen). We then generated cDNA from 1 µg of RNA, with the RevertAid H Minus First Strand cDNA Synthesis Kit (Thermo Scientific), according to the manufacturer’s protocol.

Real-time quantitative PCR was performed with cDNA diluted 1:10, primers (0.75 µM final concentration) and the QuantiNova SYBR Green PCR Kit (Qiagen). The following primers were used: Mcl-1 (fw 5′-ATGCTTCGGAAACTGGACAT-3′; reverse (rv) 5′-TCCTGATGCCACCTTCTAGG-3′) as the target gene and GAPDH (fw 5′-AATCCCATCACCATCTTCCA-3′; rv 5′-TGGACTCCACGACGTACTCA-3′), 18 s (fw 5’-AGAAACGGCTACCACATCCA-3’; rv: 5’-CACCAGACTTGCCCTCCA-3′) and RPS13 (5’-CGAAAGCATCTTGAGAGGAACA-3’; rv: 5’-TCGAGCCAAACGGTGAATC-3’) as housekeeping genes (Sigma). PCR was performed with the Mx3005P qPCR System (Agilent Technologies).

PCR efficiency was determined with 10-fold dilutions, according to the following equation: efficiency (*E*) = 10^(–1/slope)^. Cq values were determined with the following equation: *Cq* = *Ct*xlog_2_ (*E*). The Mcl-1 expression ratio was determined as follows: $\text{Ratio}=\left({\left(2\right)}^{\Delta \mathrm{CqMcl}-1}\right)/\left({\left(2\right)}^{\Delta \mathrm{CqHK}}\right)$, where Δ*Cq* = *Cq*_untreated_ *– Cq*_treated_.

### Statistical Analysis

We analyzed the data for BL41, RAMOS, and BL2 cells in multiple *t-*tests, with Holm-Sidak correction for multiple testing, in Prism (Graphpad Prism, RRID:SCR_002798). Data for tonsillar B cells were analyzed by paired *t*-test/within-subject analysis, in InVivoStat.

## Results

### Infiltrating B Cells Expressing Mcl-1 Are Observed in Kidney Grafts Displaying Chronic Antibody-Mediated Rejection

**Figure 1:**
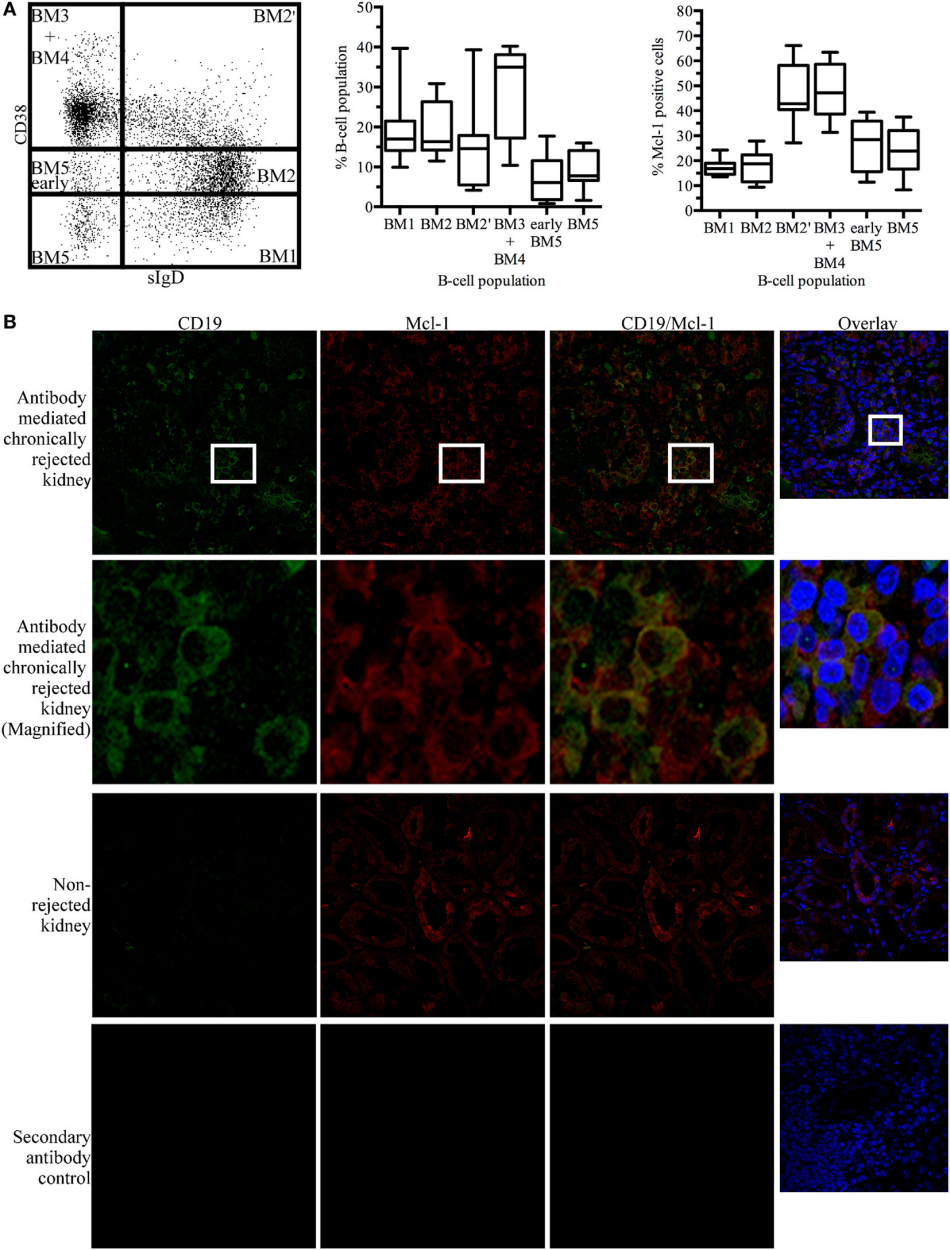
Myeloid cell leukemia-1 (Mcl-1)-expressing B cells are found in kidney grafts in cases of chronic antibody-mediated rejection **(A)** Unstimulated tonsillar cells were stained for CD19, sIgD, CD38, and Mcl-1 and analyzed by flow cytometry. B-cell populations were identified according to the BM1-BM5 classification, based on the expression of CD19, sIgD, and CD38, and additional staining for Mcl-1 was performed. The boxplot shows the distribution between donors and the quadrant indicates the values between the fifth and ninety-fifth percentiles, *n* = 7. **(B)** Immunofluorescence staining of paraffin-embedded chronically rejected kidney with anti-CD19 and anti-Mcl-1 antibodies. Images were obtained with a Leica confocal microscope fitted with a 63× objective. sIgD, surface IgD; CD, cluster of differentiation; BM, mature B cell.

Myeloid cell leukemia-1 has been shown to be important for GC maintenance and B-cell differentiation (27). According to the mature B-cell (BM) 1-BM5 classification [expression of surface IgD (sIgD) and CD38 (31)], we determined Mcl-1 expression profiles for different B-cell populations based on the expression of sIgD and CD38: sIgD^+^CD38^−/low^ cells are naïve B cells that are undifferentiated or in the early stages of differentiation (BM1-BM2), sIgD^+^CD38^high^ cells are pre-GC cells (BM2’), sIgD^−^CD38^high^ cells are GC cells (BM3 + BM4), and sIgD^−^CD38^−/low^ cells are terminally differentiated B cells (early BM5-BM5). In tonsillar B cells, Mcl-1 levels were high in the early GC and GC populations (Figure 1A).

In kidney grafts displaying cAMR, infiltrating B cells were detected by the immunostaining of histological sections for CD19. Counterstaining for Mcl-1 revealed the colocalization of this protein with CD19. Histological sections of non-rejected human kidneys were stained in the same way, and no infiltrating B cells were observed on sections of these kidneys. However, Mcl-1 is not exclusive to B cells and was also observed in tubular structures (Figure 1B). A flow cytometry analysis of mononuclear cells isolated from kidneys detransplanted due to cAMR revealed the presence of a large proportion of infiltrating memory B cells (CD19^+^CD27^+^) and plasma cells (CD138^+^) (Figure S1 in Supplementary Material).

These results indicate that cAMR is associated with an infiltration of differentiated B cells into the kidney graft and that some of the B cells infiltrating the graft express Mcl-1, like GC cells.

### Inhibiting SYK Activity Decreases Viability and Mcl-1 Protein Levels in Germinal Center-Like Cells

**Figure 2:**
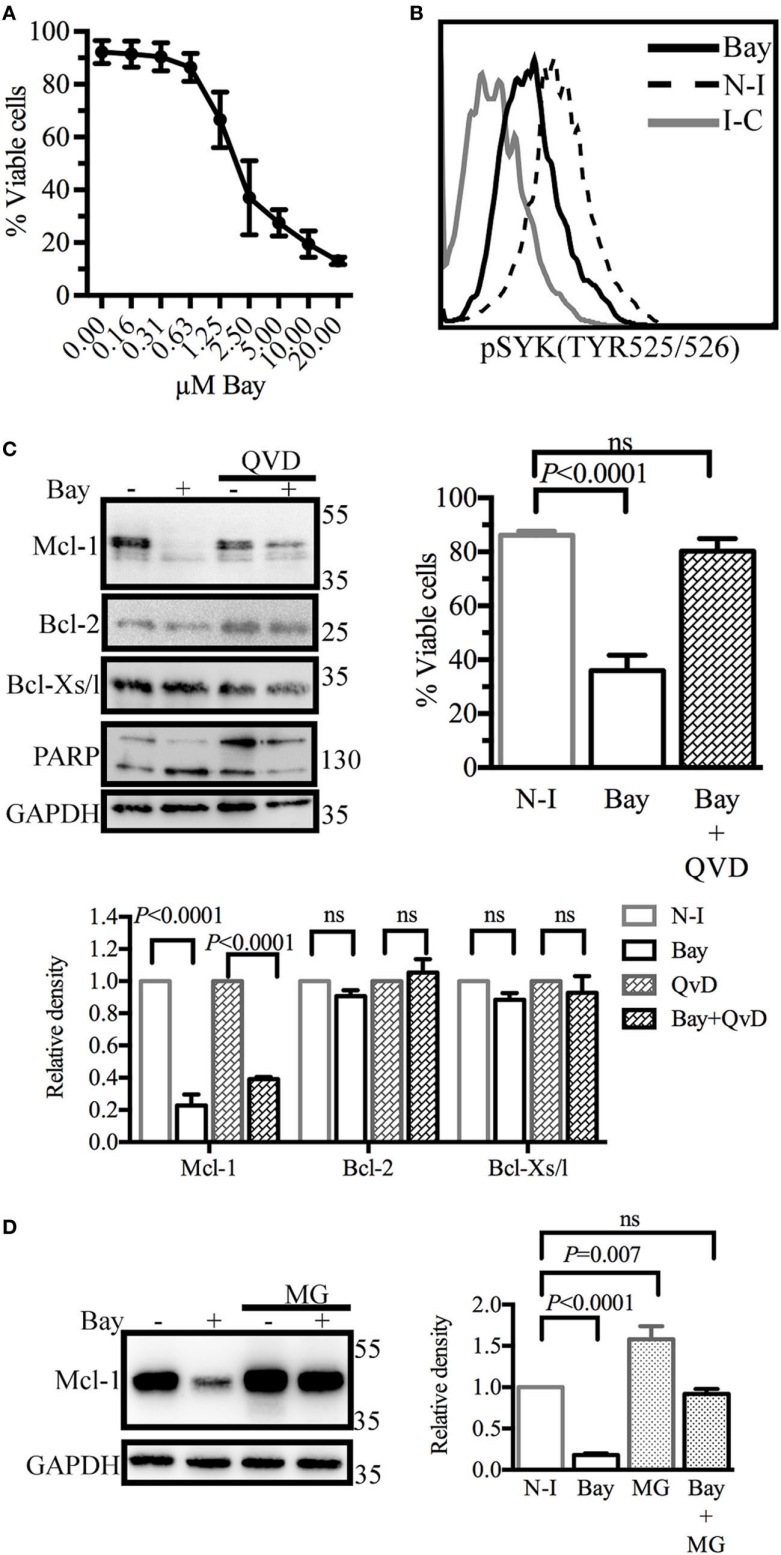
Spleen tyrosine kinase (SYK) inhibition in BL41 cells (Burkitt’s lymphoma cells) reduces viability and decreases levels of the antiapoptotic protein myeloid cell leukemia-1 (Mcl-1). **(A)** Cell viability was determined by flow cytometry analyses of cell morphology (forward and side scatter) following dose-gradient treatment with BAY61-3606 for 16 h. The data shown are mean values (±SEM, *n* = 3). **(B)** The phosphorylation status of SYK was determined by flow cytometry following SYK inhibition with BAY61-3606 (5 µM; 20 min). **(C)** BL41 cells were treated with BAY61-3606 (5 µM; 4 h) and Q-VD-Oph (10 µM; 4 h). Levels of the antiapoptotic proteins Mcl-1, B-cell lymphoma-2 (Bcl-2) and Bcl-xl were determined by western blotting, with quantification by protein densitometry and normalization against GAPDH levels. The data shown are mean values (±SEM, *n* = 3). Q-VD-Oph efficiency was determined by western-blot analysis of PARP cleavage (4 h) and cell death inhibition (16 h). The data shown are mean values (±SEM, *n* = 5). **(D)** BL41 cells were treated with BAY61-3606 (5 µM; 4 h) and MG-132 (10 µM; 4 h) and Mcl-1 protein levels were determined by western blotting, with quantification by protein densitometry and normalization against GAPDH levels. The data shown are mean values (±SEM, *n* = 5). Bay, BAY61-3606; N-I, non-inhibited; I-C, isotype control; QVD, Q-VD-Oph; ns, not significant; MG, MG132.

We then investigated ways of disrupting GC B-cell responses. Given the key role of SYK downstream from the BCR, we treated BL41 cells, a Burkitt’s lymphoma cell line used as a model of GC centroblasts (32), with the SYK inhibitor BAY61-3606 (33), to inhibit BCR signaling. We assessed the effect of SYK inhibition on cell viability. Shrinkage and blebbing are two characteristic features of apoptotic cells (34). We therefore identified dead cells on the basis of their small size and high granularity. Cell viability was decreased in a dose-dependent manner by BAY61-3606 treatment, with a 70% decrease observed in the presence of 5 µM BAY61-3606 (Figure 2A). We confirmed that BAY61-3606 reduced the level of SYK phosphorylation at TYR525/526, which is located in the activation loop of the kinase (11), in unstimulated BL41 cells (Figure 2B).

We then analyzed the levels of the antiapoptotic proteins Mcl-1, Bcl-2, and Bcl-xl. SYK inhibition resulted in lower levels of Mcl-1 protein, whereas the levels of the Bcl-2 and Bcl-xl proteins were unaffected. Mcl-1 may be cleaved by activated caspases (35) or degraded by the proteasome (36). We assessed the possible caspase-dependent degradation of Mcl-1, by treating cells with the caspase inhibitor Q-VD-Oph and BAY61-3606. This treatment resulted in only a partial rescue of Mcl-1 levels, but with the successful inhibition of caspase activation and the induction of cell death, as shown by FACS analysis and the cleavage pattern of PARP, a known substrate of caspase-3 (Figure 2C). Similar profiles for Mcl-1 and viability following SYK inhibition in the presence of Q-VD-Oph were observed with the Burkitt’s lymphoma cell lines RAMOS and BL2 (Figure S2A in Supplementary Material).

The inhibition of proteasome activity by MG132 led to an increase in Mcl-1 protein levels in control cells. However, Mcl-1 levels in cells treated with BAY61-3606 and MG132 remained similar to those in control cells (Figure 2D), suggesting that SYK inhibition does not modify the rate of Mcl-1 protein degradation. RAMOS and BL2 cells treated with BAY61-3606 and MG132 had similar Mcl-1 expression profiles (Figure S2B in Supplementary Material).

### SYK Inhibition Modulates Mcl-1 Gene Expression and Alters the Cellular Distribution of STAT3

**Figure 3:**
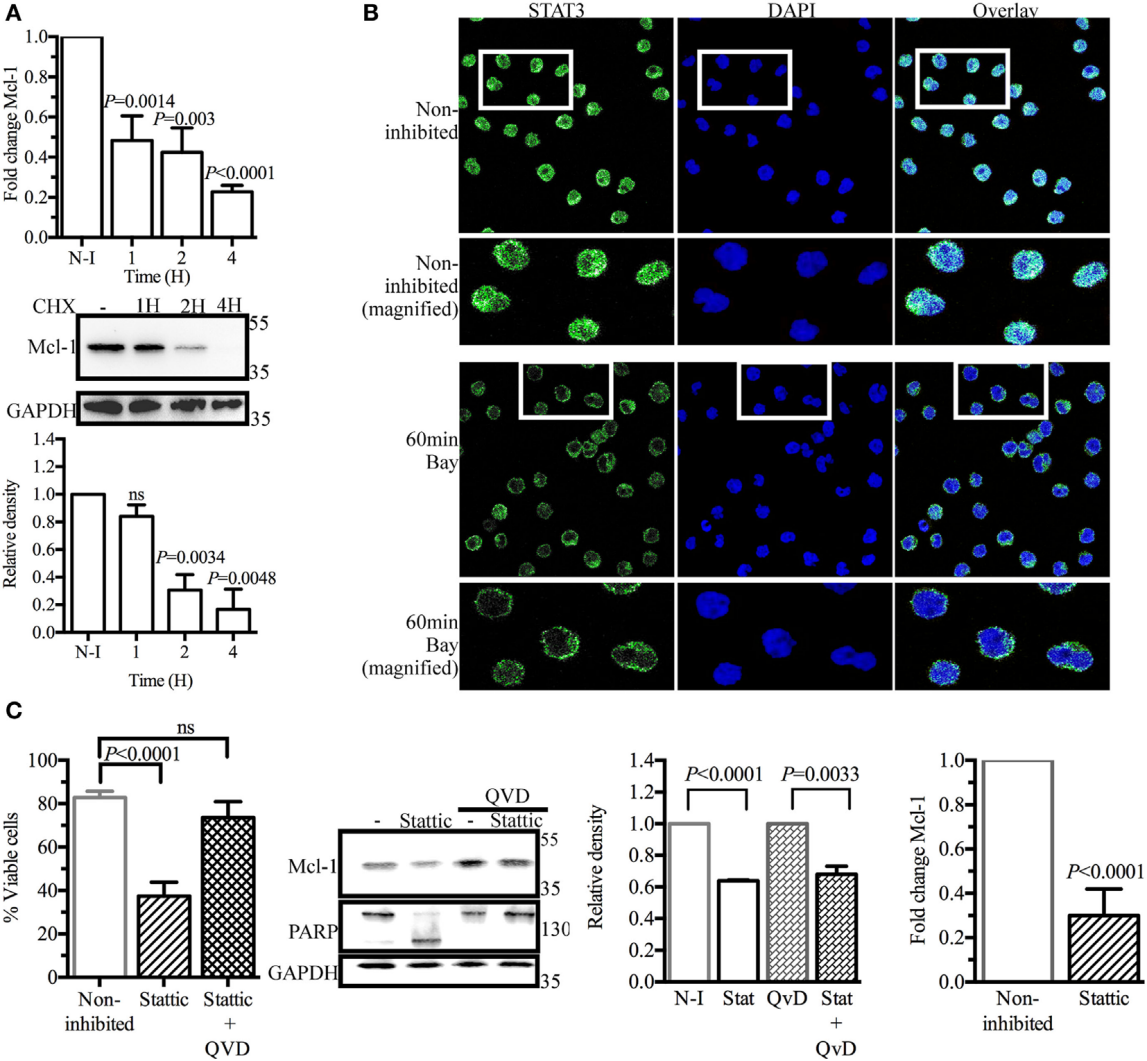
Signal transducer and activator of transcription 3 (STAT3) as a potential regulator of myeloid cell leukemia-1 (Mcl-1) gene transcription in BL41 cells (Burkitt’s lymphoma cells) upon spleen tyrosine kinase inhibition. **(A)** BL41 cells were treated with BAY61-3606 (5 µM) for 1, 2, and 4 h and expression of the Mcl-1 gene was assessed by RT-qPCR. Mean values are shown (±SEM, *n* = 5), and significant *P*-values are indicated for comparisons with cells not treated with BAY61-3606. BL41 cells were treated with cycloheximide (10 µM) for 1, 2, and 4 h and Mcl-1 protein levels were determined by western blotting, with quantification by protein densitometry and normalization against GAPDH levels. Mean values are shown (±SEM, *n* = 3), and significant *P*-values are indicated for comparisons with cells not treated with BAY61-3606. **(B)** BL41 cells were treated with BAY61-3606 (5 µM; 60 min), and the cellular location of STAT3 was determined by immunofluorescence analyses. Images were obtained with a Leica confocal microscope equipped with a 63× objective. **(C)** The viability of BL41 cells was determined by flow cytometry analysis in cells treated with the STAT3 inhibitor Stattic (5 µM; 16 h) in the presence or absence of Q-VD-Oph (10 µM). Apoptotic cells were identified on the basis of their size and granularity, as assessed by forward and side light scattering. The data shown are mean values (±SEM, *n* = 6). Mcl-1 protein levels were assessed by western blotting after treatment with Stattic (5 µM; 4 h) and Q-VD-Oph (10 µM); Mcl-1 levels were quantified by protein densitometry and normalized against GAPDH levels. The data shown are mean values (±SEM, *n* = 3). Q-VD-Oph efficiency was determined by western blotting to analyze PARP cleavage (4 h) and cell death inhibition (16 h). Mean values are shown (±SEM, *n* = 6). Mcl-1 gene expression levels were determined by RT-qPCR. Mean values are shown (±SEM, *n* = 4), and significant *P*-values are shown for comparisons with cells not treated with BAY61-3606. CHX, cycloheximide; N-I, non-inhibited; Bay, BAY61-3606; QVD, Q-VD-Oph; ns, not significant.

As SYK inhibition did not accelerate Mcl-1 protein degradation in BL41 cells, we hypothesized that the lower levels of Mcl-1 protein observed in BL41 cells exposed to BAY61-3606 might result from lower levels of *de novo* protein synthesis. We tested this hypothesis by determining Mcl-1 mRNA levels by RT-qPCR. BAY61-3606 treatment substantially decreased Mcl-1 gene expression in BL41 cells, for up to 4 h after treatment. Lower levels of Mcl-1 mRNA were also observed in RAMOS and BL2 cells exposed to BAY61-3606 (Figure S3A in Supplementary Material). The short turnover of the Mcl-1 protein in BL41 cells was confirmed by blocking protein synthesis with cycloheximide, which resulted in lower levels of Mcl-1 protein after 2 h, supporting the hypothesis that SYK inhibition affects *de novo* protein synthesis (Figure 3A).

Signal transducer and activator of transcription 3 (Stat3) has been identified as a major regulator of Mcl-1 gene transcription (37, 38). Immunohistological analysis in basal conditions showed STAT3 to be present in the nucleus of BL41 cells, where it can mediate Mcl-1 gene transcription. BAY61-3606 inhibited the translocation of STAT3 from the cytoplasm to the nucleus (Figure 3B), suggesting a role for STAT3 in the regulation of Mcl-1 gene expression by BCR signaling. Incubation with the STAT3 inhibitor Stattic resulted in a 70% decrease in cell viability associated with the lower levels of Mcl-1 protein. Caspase inhibition by Q-VD-Oph rescued the cells from the cell death induced by Stattic, whereas Mcl-1 protein levels were only partially rescued, suggesting that STAT3 inhibition does not result in a caspase-dependent cleavage of Mcl-1 similar to that observed with BAY61-3606. The lack of PARP cleavage confirmed the effectiveness of Q-VD-Oph, as shown in Figure 2C. Similarly, Stattic treatment decreased Mcl-1 protein levels in both RAMOS and BL2 cells. Caspase inhibition with Q-VD-Oph in the presence of Stattic did not affect Mcl-1 levels, as already observed in BL41 cells (Figure S3B in Supplementary Material). We assessed Mcl-1 gene expression by RT-qPCR. We found that 4 h of treatment with Stattic decreased the level of Mcl-1 gene expression (Figure 3C). Treatment with Stattic in RAMOS and BL2 cells also decreased Mcl-1 transcript levels (Figure S3B in Supplementary Material).

Taken together, these results indicate that STAT3 is involved in regulating Mcl-1 gene expression in BL41, RAMOS, and BL2 cells.

### Overexpression of the Mcl-1 Gene Counteracts the Inhibition of Both SYK and STAT3

**Figure 4:**
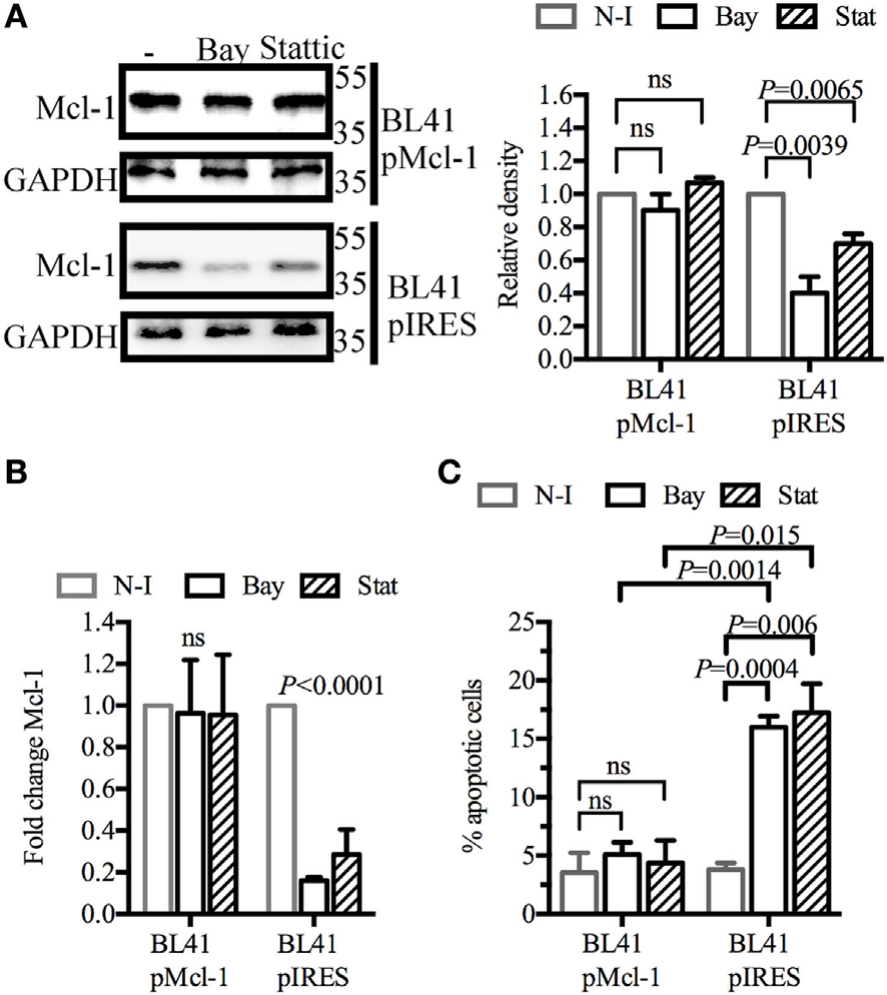
Myeloid cell leukemia-1 (Mcl-1) overexpression protects BL41 cells against BAY61-3606-induced apoptosis BL41 cells were transduced with retroviral particles containing an Mcl-1 construct (pMcl-1), or an empty vector (pIRES) as a control. **(A)** The transduced cells were treated with BAY61-3606 or Stattic (5 µM; 4 h). Mcl-1 protein levels were determined by western blotting, with quantification by protein densitometry and normalization against GAPDH levels. Mean values are shown (±SEM, *n* = 3). **(B)** Mcl-1 gene expression was determined by RT-qPCR after treatment with BAY61-3606 (5 µM; 4 h) or Stattic (5 µM; 4 h). Mean values are shown (±SEM, *n* = 4), and significant *P*-values are shown for comparisons with cells not treated with inhibitor. **(C)** Apoptotic cells were identified on the basis of their extracellular expression of phosphatidylserine, detected by Annexin V staining, and their plasma membrane permeability, assessed by monitoring their uptake of 7-AAD after treatment with BAY61-3606 (5 µM; 8 h) or Stattic (5 µM; 8 h). Mean values are shown (±SEM, *n* = 4). Bay, BAY61-3606; N-I, non-inhibited; ns, not significant; Stat, Stattic.

We investigated whether Mcl-1 gene overexpression could rescue BL41 cells from apoptosis. Cells were transduced with retroviral particles containing an Mcl-1 construct, or with control particles lacking this construct. As expected, the Mcl-1 overexpression induced by retroviral particles was not influenced by BAY61-3606 or Stattic in transduced BL41 cells (Figure 4A). A similar effect was observed for levels of Mcl-1 gene expression (Figure 4B). The effect of BAY61-3606 and Stattic on apoptosis was then assessed in both types of BL41 cells, in a flow cytometry assay based on the extracellular expression of phosphatidylserine and 7-AAD uptake. The proportion of apoptotic cells increased in cells transduced with the empty vector, whereas no such increase upon SYK or STAT3 inhibition was observed in cells overexpressing Mcl-1 (Figure 4C). We conclude that the downregulation of Mcl-1 in BL41 cells following SYK inhibition is required to induce apoptotic cell death.

### SYK Inhibition in Tonsillar B Cells Decreases Viability, Activation, and Mcl-1 Expression, Resulting in Lower Levels of Ig Secretion

**Figure 5:**
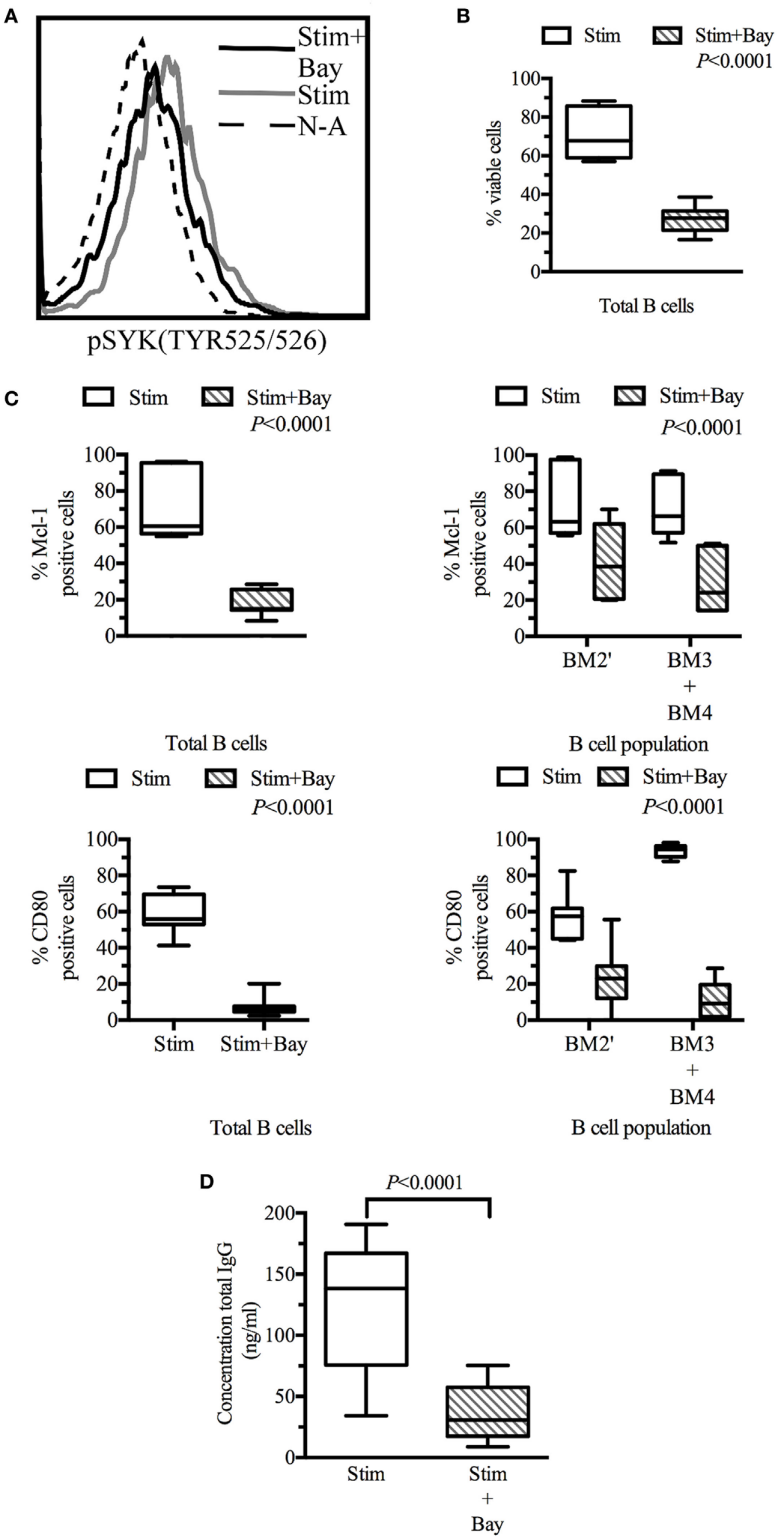
Spleen tyrosine kinase (SYK) inhibition in tonsillar B cells decreases viability, activation, and effector functions. Tonsillar cells activated *in vitro* by incubation with CD40L fibroblasts (1:10 ratio, fibroblasts: tonsillar cells), anti-μ antibody (10 µg/ml) and LPS (1 µg/ml) Non-activated tonsillar cells were cultured with CD32L fibroblasts (1:10 ratio, fibroblasts: tonsillar cells). **(A)** Following incubation for 20 min in the presence or absence of BAY61-3606 (5 µM), B cells were identified on the basis of their expression of CD19, and SYK phosphorylation status was determined by flow cytometry. **(B)** Following 3 days of culture, cells were stained with a fixable viability stain and identified on the basis of their CD19 expression status, by flow cytometry. Dead B cells were identified with a fixable viability stain. The boxplot shows the distribution between donors, and the quadrant indicates the values between the fifth and ninety-fifth percentiles, *n* = 7. **(C)** Following 3 days of culture, cells were stained with fixable viability stain, and for CD19, sIgD, CD38, CD80, and myeloid cell leukemia-1 (Mcl-1), and analyzed by flow cytometry. Viable cells were identified as fixable viability stain-negative. Mcl-1 levels were determined in CD19-positive cells and in pre-GC (BM2′) and GC B cells (BM3 + BM4) identified on the basis of sIgD and CD38 expression profiles. Activation state was determined by assessing CD80 expression in CD19-positive cells and in pre-GC (BM2′) and GC B-cells (BM3 + BM4). The boxplot represents the distribution between donors and the quadrant indicates the values between the fifth and ninety-fifth percentiles; significant *P* values are shown on the graphs for comparisons between all stimulated cells and those treated with BAY61-3606, *n* = 7. **(D)** The total immunoglobulin G (IgG) secreted by the cells during 3 days of culture was analyzed by ELISA. The boxplot represents the distribution between donors and the quadrant indicates the values between the fifth and ninety-fifth percentiles, *n* = 9. stim, stimulated; Bay, BAY61-3606; N-A, non-activated; CD, cluster of differentiation.

We then sought to confirm the general effect of SYK inhibition on primary B cells activated *in vitro*. An analysis of SYK phosphorylation revealed that BAY61-3606 reduced the phosphorylation state of SYK in activated B cells (Figure 5A).

Flow cytometry analyses were performed on tonsillar B cells (CD19^+^) after 3 days in culture. The viability of activated B cells in which BCR signaling was impaired by BAY61-3606 was 60% lower than that of control cells. Non-activated cells were not viable in culture, and no difference was observed between non-activated cells in the presence and absence of Bay61-3606 (Figure 5B).

Lower B-cell viability was associated with lower levels of Mcl-1 protein and of B-cell activation, as assessed by monitoring CD80 expression. This effect was observed in total B cells and in the pre-GC and GC (BM2′ and BM3 + BM4) populations identified on the basis of the BM1–BM5 classification (Figure 5C).

We then investigated the effects of SYK inhibition on Ig production, by assessing IgG secretion after 3 days in culture. Activated cells secreted about 150 ng of IgG per ml, whereas activated cells treated with BAY61-3606 secreted smaller amounts of IgG (Figure 5D).

These results suggest that SYK inhibition reduces cell viability and Mcl-1 protein levels and impairs B-cell activation and IgG secretion.

## Discussion

The GC reaction is important for the generation of effector B cells with a high Ag affinity. During the GC reaction, apoptosis is required to eliminate B cells with low Ag affinity and self-reactive B cells (19, 39, 40).

Conversely, the positive selection of GC B cells with a high Ag affinity and the promotion of their survival are essential for the generation of effector B cells (19, 20). The role of antiapoptotic Bcl-2 family members, including Bcl-2, Mcl-1, and Bcl-xl, in the GC reaction has been investigated. Yoshino et al. (41) observed that resting and mantle-zone B cells expressed Bcl-2, whereas GC B cells did not. This finding was confirmed by the identification of Bcl-6 as a repressor of Bcl-2 transcription (42, 43). We confirmed, in a previous study based on the *in situ* staining of human lymphocytes, that GC cells do not express Bcl-2, whereas Mcl-1 is co-expressed with PUMA and Bcl-xl in these cells (28). There is growing evidence to suggest that Mcl-1 is a key player in the survival of activated B cells. Vikstrom et al. (27) showed, with a conditional Mcl-1 knockout model in mice, that the absence of Mcl-1 resulted in the defective development of GC and memory cells and impaired Ig secretion. In the same study, memory B-cell formation had no effect on the GC reaction in Bcl-xl knockout mice. We confirm here that Mcl-1 is strongly expressed in the BM3 + BM4 population of tonsillar B cells corresponding to GC B cells (31).

A recent study showed that GCs may develop outside secondary lymphoid organs and form TLS, which generate effector and memory B cells. Such TLS have been reported in cases of persistent inflammation (44, 45). Thaunat et al. (14) detected TLS in kidney allografts in cases of cAMR, in which infiltrating B cells, T cells, and follicular dendritic cells were identified. GC B-cell characteristics, such as clonality, high levels of proliferation, and the upregulation of GC-related genes, have been observed in these infiltrations (14, 46–48). We found that the B cells infiltrating kidneys displaying cAMR expressed Mcl-1, consistent with the notion that the B cells infiltrating grafted organs are part of the TLS and are involved in the local immune response and effector B-cell production. Various immunosuppressive treatments have been developed to prevent and treat acute cellular rejection, but these treatments are clearly ineffective against cAMR, which remains a leading cause of chronic organ failure (49–51). There is, therefore, an urgent need to develop new treatments that effectively alter GC-like cell viability and differentiation, to reduce the prevalence of cAMR.

B-cell receptor signaling during B-cell development leads to the elimination of self-reactive B cells by programmed cell death (52, 53), whereas Ag affinity-linked BCR engagement later in the GC reaction leads to the apoptosis of cells with a low Ag affinity and the differentiation of cells with a high Ag affinity into effector B cells (19–21). SYK acts very early in the signaling chain, and is, therefore, an attractive candidate regulator of BCR signaling. In this study, we aimed to impair BCR signaling by inhibiting the kinase activity of SYK, to decrease GC B-cell viability. In GC centroblast cell lines, inhibition of the constitutively active SYK kinase resulted in the downregulation of Mcl-1 and a decrease in cell viability. We show that preventing Mcl-1 protein degradation with caspase or proteasome inhibitors only partially rescues Mcl-1 protein levels, and our RT-qPCR experiments confirmed the downregulation of Mcl-1 gene transcription.

Akgul et al. (54) identified several binding sites for transcription factors, including STAT3, within the promotor region of the Mcl-1 gene. The role of STAT3 in the GC response remains unclear, but this transcription factor has been shown to influence the T cell-dependent IgG response, and STAT3 deficiencies impair the generation of human memory B cells (55, 56). Ding et al. (57) recently showed, with a STAT3 knockout mouse model, that STAT3 is dispensable for GC initiation but essential for the maintenance of the GC reaction. Immunized STAT3 knockout mice had larger numbers of apoptotic GC B cells than normal mice, and this higher level of apoptosis was associated with lower levels of Mcl-1 gene expression (57). STAT3 has already been reported to be involved in Mcl-1 regulation, but mostly on the basis of observations from experiments in which the JAK/STAT pathway was activated by cytokine- and growth factor-induced signaling (58–60). We show here, in BL41 cells, that SYK inhibition impairs the translocation of STAT3 to the nucleus, preventing its binding to the promoter of the Mcl-1 gene, whereas this transcription factor remains constitutively nuclear in the absence of treatment. STAT3 inhibition mimicked the effects of SYK inhibition in terms of Mcl-1 expression and cell death. We show here that BCR signaling is associated with the STAT3-modulated regulation of Mcl-1 protein levels. The modulation of STAT3 levels by the BCR is supported by data from studies reporting a JAK-independent link between BCR signaling and STAT3 (61, 62). We also show that Mcl-1 overexpression prevents cells from entering apoptosis in the presence of inhibitors of both SYK and STAT3. These results suggest that Mcl-1 downregulation is required to induce apoptosis, confirming the key role of Mcl-1 in maintaining the GC reaction, as reported in previous studies (27, 28, 63).

Le Huu et al. (64) observed an increase in SYK phosphorylation in B cells following allogeneic bone marrow transplantation in mice. In human patients with graft versus host disease (GVDH), total SYK levels were found to be higher than those in healthy patients. In the same study, PBMCs isolated from GVDH patients were found to have a higher BCR responsiveness following BCR engagement than PBMCs from healthy patients. SYK inhibition reduced BCR responsiveness in these cells (65). These findings suggest that activated SYK plays a role in GVDH. We show here that SYK inhibition in activated B cells reduces cell viability *in vitro*. Our results are consistent with those of Flynn et al. (66), showing that SYK inhibition induces apoptosis more strongly in B cells isolated from the PBMCs of patients with active GVDH than in those from patients with inactive or no GVDH. We found that this lower viability was associated with Mcl-1 downregulation in BL41 cells, a finding confirmed by our experiments with primary tonsillar B cells. We also show that SYK inhibition is associated with weaker activation, as shown by the level of CD80 expression.

The damaging role of the DSAs produced following organ transplantation has been studied in detail. Preexisting and *de novo* IgG DSAs are associated with acute and chronic solid graft injury (51, 67, 68), whereas DSAs of the IgM and IgA types are not in themselves damaging (69, 70). We showed that SYK inhibition in primary cells activated *in vitro* abolished all IgG secretion. These results therefore suggest that SYK inhibition affects the GC reaction and GC B-cell responsiveness.

In conclusion, our data show that SYK inhibition affects BCR signaling-mediated cell survival through the downregulation of Mcl-1 gene transcription. We demonstrate that SYK inhibition impairs B-cell responses by altering B-cell reactivity to Ags and decreasing Ab production. These results suggest that, in cases of cAMR, SYK could be targeted by new therapeutic tools, to improve graft survival by manipulating the humoral immune response.

## Ethics Statement

Renal biopsy specimens and detransplanted kidneys were obtained from transplant recipients with end-stage renal failure due to cAMR, who gave written informed consent in accordance with the Declaration of Helsinki for the use of part of their biopsy specimens for research on renal transplantation. The samples were stored at the Biological Resource Center of Paris-South University and the collection has been declared to the French Ministry of Research: AC-2017-2991. The tonsil used were collected 5 years ago from children with tonsillar hypertrophy. Tonsils removed from children in this context are generally considered to be waste and are normally immediately destroyed in the operating room without histological analysis. We have such tonsils, before their destruction. The project and the protocol were submitted to the ED468 (Biosigne) doctoral school for approval.

## Author Contributions

AD, AV, and NR designed the study; NR, FH, GA, and AP performed experiments; FH and GA contributed to study design; OT helped to obtain histological slides for cAMR kidney samples; NR prepared the manuscript; and the other authors commented on the manuscript.

## Conflict of Interest Statement

The authors declare that the research was conducted in the absence of any commercial or financial relationships that could be construed as a potential conflict of interest.

## Supplementary Material

Figure S1 Unstimulated B-cells isolated from mononuclear cells extracted from transplant recipients presenting end-stage renal failure due to chronic AMR were stained for CD19, CD27 and CD138 and analyzed by flow cytometry. Memory B-cells were identified based on the expression of CD19 and CD27. Plasma cells were identified based on the expression of CD138, *n* = 4.

Figure S2 **(A)** BL2 and RAMOS cells were treated with BAY61-3606 (5 µM; 4 h) and Q-VD-Oph (10 µM; 4 h). Levels of the antiapoptotic proteins myeloid cell leukemia-1 (Mcl-1), were determined by western blotting. Q-VD-Oph efficiency was determined by western-blot analysis of PARP cleavage (4 h) and cell death inhibition (16 h). The data shown are mean values (±SEM, *n* = 3). **(B)** BL2 and RAMOS cells were treated with BAY61-3606 (5 µM; 4 h) or MG-132 (10 µM; 4 h) and Mcl-1 protein levels were determined by western blotting, with quantification by protein densitometry and normalization against GAPDH levels (*n* = 1). Bay: BAY61-3606, N-I: non-inhibited, QVD: Q-VD-Oph, MG: MG132.

Figure S3 **(A)** BL2 and RAMOS cells were treated with BAY61-3606 (5 µM; 4 h) and expression of the myeloid cell leukemia-1 (Mcl-1) gene was assessed by RT-qPCR. Mean values are shown (±SEM, *n* = 3). **(B)** Mcl-1 protein levels were assessed by western blotting after treatment with Stattic (5 µM; 4 h) and Q-VD-Oph (10 µM). Q-VD-Oph efficiency was determined by western blotting to analyze PARP cleavage (4 h) Mcl-1 gene expression levels were determined by RT-qPCR. Mean values are shown (±SEM, *n* = 3). Bay: BAY61-3606, QVD: Q-VD-Oph.
