# Supplementary figures and images for: SYK Inhibition Induces Apoptosis in Germinal Center-Like B Cells by Modulating the Antiapoptotic Protein Myeloid Cell Leukemia-1, Affecting B-Cell Activation and Antibody Production
Source: Front Immunol. 2018 Apr 24;9:787. doi: 10.3389/fimmu.2018.00787 (PMC5928208; doi:10.3389/fimmu.2018.00787)

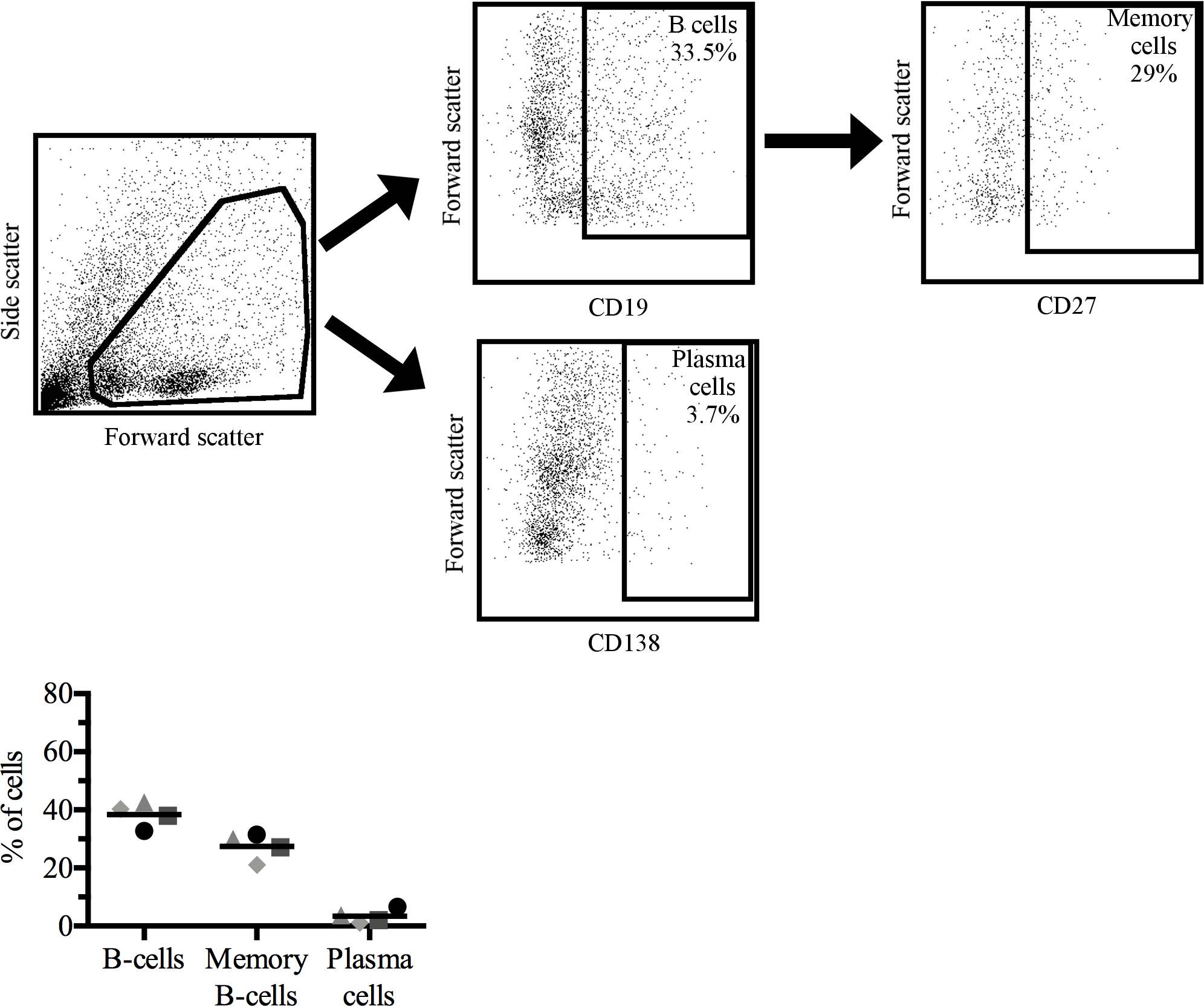

Supplement: Figure S1 — Unstimulated B-cells isolated from mononuclear cells extracted from transplant recipients presenting end-stage renal failure due to chronic AMR were stained for CD19, CD27 and CD138 and analyzed by flow cytometry. Memory B-cells were identified based on the expression of CD19 and CD27. Plasma cells were identified based on the expression of CD138, n = 4. [file image_1.tiff]

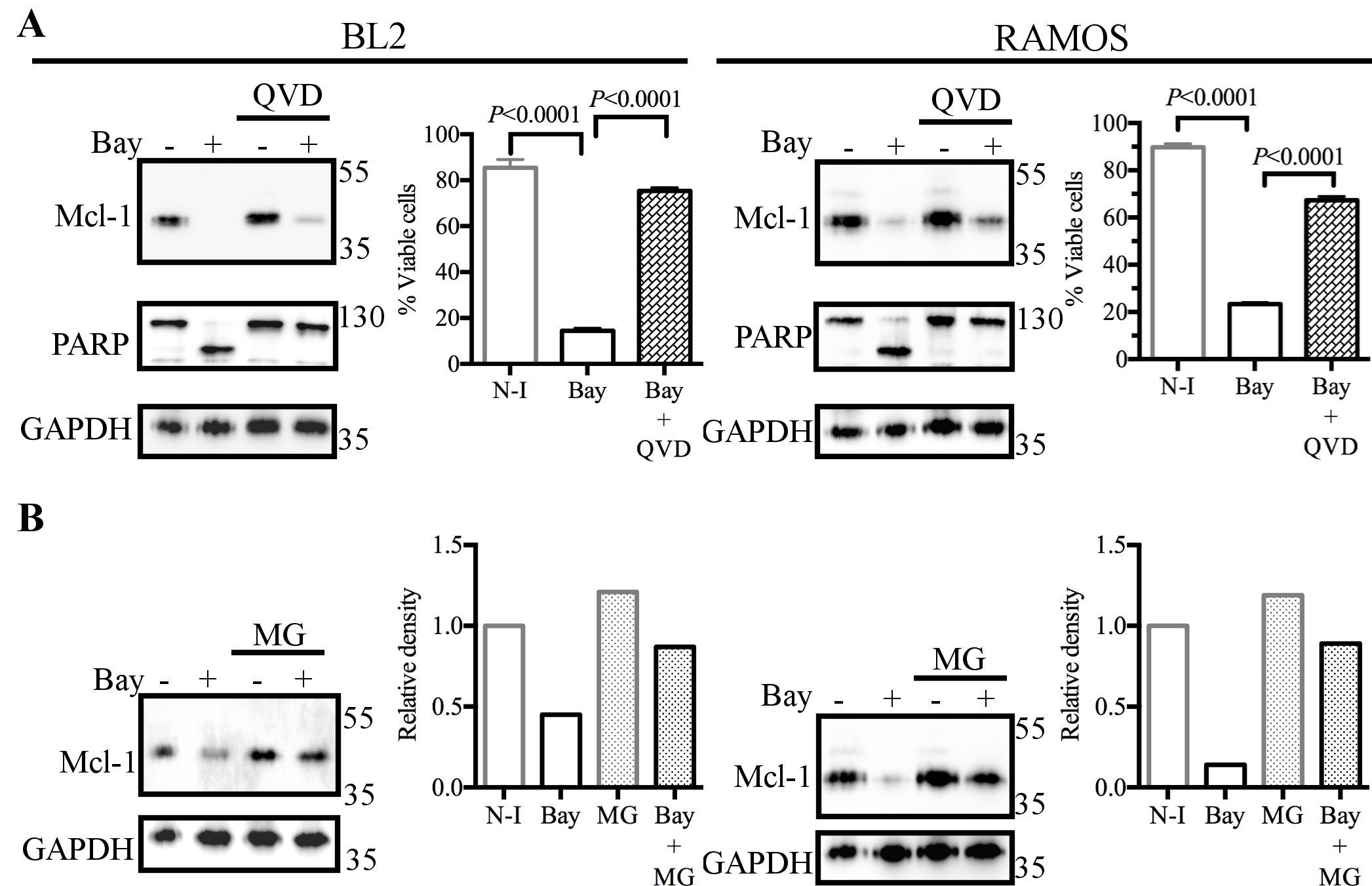

Supplement: Figure S2 — (A) BL2 and RAMOS cells were treated with BAY61-3606 (5 µM; 4 h) and Q-VD-Oph (10 µM; 4 h). Levels of the antiapoptotic proteins myeloid cell leukemia-1 (Mcl-1), were determined by western blotting. Q-VD-Oph efficiency was determined by western-blot analysis of PARP cleavage (4 h) and cell death inhibition (16 h). The data shown are mean values (±SEM, n = 3). (B) BL2 and RAMOS cells were treated with BAY61-3606 (5 µM; 4 h) or MG-132 (10 µM; 4 h) and Mcl-1 protein levels were determined by western blotting, with quantification by protein densitometry and normalization against GAPDH levels (n = 1). Bay: BAY61-3606, N-I: non-inhibited, QVD: Q-VD-Oph, MG: MG132. [file image_2.tiff]

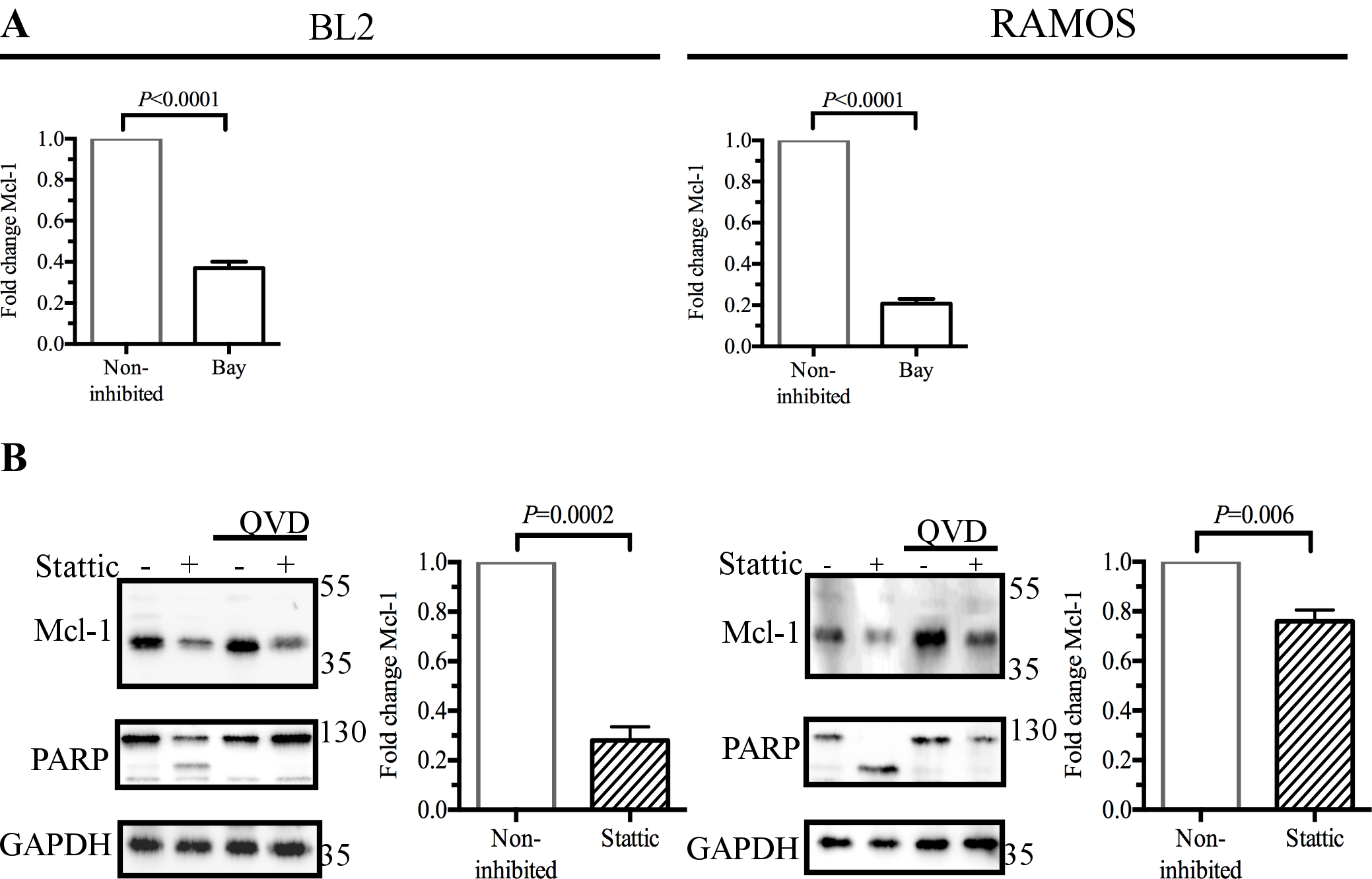

Supplement: Figure S3 — (A) BL2 and RAMOS cells were treated with BAY61-3606 (5 µM; 4 h) and expression of the myeloid cell leukemia-1 (Mcl-1) gene was assessed by RT-qPCR. Mean values are shown (±SEM, n = 3). (B) Mcl-1 protein levels were assessed by western blotting after treatment with Stattic (5 µM; 4 h) and Q-VD-Oph (10 µM). Q-VD-Oph efficiency was determined by western blotting to analyze PARP cleavage (4 h) Mcl-1 gene expression levels were determined by RT-qPCR. Mean values are shown (±SEM, n = 3). Bay: BAY61-3606, QVD: Q-VD-Oph. [file image_3.tiff]
